# Epidemiological and Clinical Characteristics of 217 COVID-19 Patients in Northwest Ohio, United States

**DOI:** 10.7759/cureus.14308

**Published:** 2021-04-05

**Authors:** Mamtha Balla, Ganesh Merugu, Zeid Nesheiwat, Mitra Patel, Taha Sheikh, Rawish Fatima, Vinay K Kotturi, Venugopal Bommana, Gautham Pulagam, Brian Kaminski

**Affiliations:** 1 Internal Medicine, ProMedica Toledo Hospital, Toledo, USA; 2 Internal Medicine, The University of Toledo Medical Center, Toledo, USA; 3 Geriatrics, The University of Toledo Medical Center, Toledo, USA; 4 Internal Medicine, The University of Toledo College of Medicine, Toledo, USA; 5 Family Medicine, The University of Toledo Medical Center, Toledo, USA; 6 Internal Medicine, Medical University of the Americas, Charlestown, KNA; 7 Emergency Medicine, ProMedica Toledo Hospital, Toledo, USA

**Keywords:** covid-19, epidemiological findings, clinical findings, predictors of mortality, northwest ohio

## Abstract

Background

There is limited data on the clinical characteristics and predictors of mortality of coronavirus disease-2019 (COVID-19) in North West Ohio. We performed a retrospective review of patients hospitalized with COVID-19 in the ProMedica Health System in Northwest Ohio from March 25 to June 16, 2020. The study aims to identify epidemiological, clinical characteristics, and predictors of Mortality of COVID-19 patients in Northwest Ohio.

Methods

This study was conducted on 217 COVID-19 patients admitted to ProMedica Health System Hospitals in Northwest Ohio from March 25 to June 16, 2020. We collected data, including clinical signs, symptoms, and outcomes of the COVID-19 patients. We compared clinical signs and symptoms along with comorbidities of survivors and non-survivors.

Results

Of the 217 patients included in the study, the mean age of the population was 63.13 (SD 17.8), of which 194 (89.4%, mean age 61.7 years) survived while 23 (10.6%, mean age 74.6 years) died. Among them, 53% were females and 47% male. Common presenting symptoms were chest pain (91.71%), shortness of breath (79.7%), cough (71%), and fever (64%). Mortality was associated with age greater than 63 (p-value 0.0052) and hypertension (p-value: 0.0058) with marginal significance with gender (p-value: 0.0642), chest pain (p-value: 0.0944), and history of cancer (p-value: 0.0944).

Conclusions

Advanced age and hypertension (HTN) are independent predictors for increased mortality. History of cancer and chest pain are associated with increased mortality with marginal significance. Awareness among physicians about predictors of mortality is essential in dealing with COVID-19 patients. It is essential to educate the public about preventative strategies such as wearing masks to decrease mortality and morbidity from this pandemic.

## Introduction

This article aims to provide a comprehensive analysis of the epidemiology and clinical characteristics of coronavirus disease-2019 (COVID-19) patients in Northwest Ohio. The novel coronavirus 2019 (2019 nCoV), also known as severe acute respiratory syndrome coronavirus two or COVID-19, originated in Wuhan, China when patients presented with atypical pneumonia unknown etiology. The initial human cases of COVID-19 were reported in December 2019. While it cannot be determined how humans in China were initially infected, evidence suggests that COVID-19 has a natural animal origin, and bats serve as its environmental reservoirs [1]. The initial cases suggested an animal to human transmission, and after an exponential increase in the number of cases, human to the human transmission had also become apparent, especially between close contacts. The primary mode of transmission is thought to be by respiratory droplets or by contact with infected secretions [2]. The World Health Organization declared this outbreak as a pandemic on March 11, 2020, the median incubation period is 5.1 days, with the onset of symptoms to occur within 11.5 days for 97.5% of the infected population [3]. The most common symptoms reported include fever, dry cough or chest tightness, and dyspnea; severe cases of infection can lead to pneumonia, multiple organ failure, and death [4].

There is a higher mortality rate in men than women, potentially due to sex-based immunological or gendered differences [5]. Few studies also showed higher mortality in patients with COVID-19 due to the renal involvement [6]. As of February 17, 2021, there are 824,401 confirmed cases in Ohio, with 49,788 hospitalizations, including 7,083 ICU admissions. The median age is 42, and 46% of the infected cases are males, and 53% are females [7].

Very few studies are available in the United States mid-western region to identify predictors of Mortality, clinical signs, and symptoms of COVID-19 patients. This is one of the first studies in Midwestern, especially Northwest Ohio, to identify outcomes of COVID-19 patients based on demographics, clinical signs and symptoms. As per the studies, only with a multidirectional approach, we can mitigate COVID-19 effectively in the community as false-negative cases are prevalent [8]. As of February 24, 2021, 112,138,700 people contracted and 2,486,300 died from Coronavirus [9].

## Materials and methods

This study was conducted according to ProMedica Health System guidance and approved by the ProMedica Institutional Review Board. This is a retrospective review where we analyzed anonymous clinical data on patients with positive COVID-19 by PCR. Due to the study's characteristics, where it included only retrospective review, informed consent is waived. Data regarding epidemiological, clinical signs and symptoms were analyzed from 217 patients of COVID-19 who were admitted from March 25 to June 16, 2020. The data includes demographic (age, race, gender), epidemiological, clinical features (cough, fever, shortness of breath, loss of smell, loss of taste, gastrointestinal [GI] symptoms including nausea, vomiting, diarrhea, headache) and outcome of the patients (still in the hospital, died, or discharged). Six independent reviewers extracted data from medical records and evaluated for eligibility for the study. All the data were initially entered except the collection sheet, which has inbuilt checks to ensure data is collected appropriately. To ensure an independent evaluator assessed further 15% of all the cases (approximately 45 patients) for the date's validity and consistency. Four researchers analyzed all data. A confirmed case of COVID-19 was defined as a suspected patient with a nasopharyngeal specimen, which shows the positive result by real-time reverse transcription-polymerase chain reaction assay-RT-PCR assay. We Involved all subjects admitted to the Promedica Healthcare System and diagnosed COVID-19 infection during their admission from March to June 2020. We excluded all subjects who did not have the diagnosis of COVID-19 infection and who were not admitted to ProMedica Health System were excluded. We obtained the subjects and related information from Promedica Health system inpatient records from three hospitals including Promedica Toledo Hospital, Flower Hospital, and Bay Park Hospital. Retrospective study of the data using descriptive statistics was done. Data were summarized using descriptive statistics such as means, standard deviations, medians for numerical variables. Categorical variables were identified as frequency, percentages, cumulative frequency, and cumulative percentages. Inferential statistics such as t-tests and chi-square analyses were performed where appropriate. SPSS version 22.0 (IBM Corp., Armonk, NY) was used for all analyses. All p-values were two-tailed, and a level of < 0.05 was considered significant.

## Results

We performed this study encompassing patients living in Northwest Ohio to identify comorbid risk factors, common presenting symptoms, and overall outcomes. We compared these characteristics between survivors and non-survivors and identified the most commonly associated conditions with increasing mortality.

General Information

In 217 COVID-19 PCR positive patients who were admitted to ProMedica Health System hospitals in Northwest Ohio, the mean age of the population was 63.13 (SD 17.8), of which 194 (89.4%, mean age 61.7 years) survived while 23 (10.6%, mean age 74.6 years) died. Among them, 53% were females and 47% males. Racial breakdown includes 61% white, 3% Hispanic, 35% black, and Asian 1%. 57.14% of the population were obese.

Clinical features of the study population

**Figure 1:**
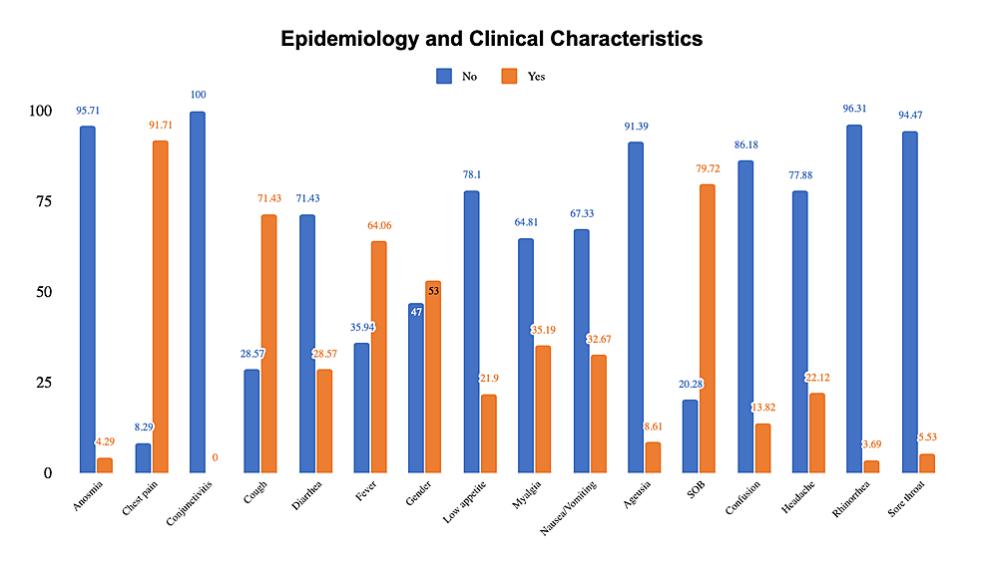
Clinical characteristics of patients admitted with COVID-19. SOB: shortness of breath.

Common presenting symptoms included chest pain (91.71%), shortness of breath (79.7%), cough (71%), fever (64%), myalgia (35%), nausea/vomiting (32.67%), headache (22 %,), and low appetite (21.9%) (Table 1, Figure 1).

**Table 1:** Clinical characteristics and comorbid conditions of patients admitted with COVID-19. *Missing data is excluded from the analysis; N = number of the patients; % = percentage. DM: diabetes mellitus; GI: gastrointestinal; NSAID: nonsteroidal anti-inflammatory drug; HTN: hypertension; CVD: cardiovascular disease; RT: respiratory tract; ACEI: angiotensin-converting enzyme inhibitor; SOB: shortness of breath.

Variables	Yes (%) (N)	No (%) (N)
Anosmia	4.29 (9)	95.71 (201)*
Chest pain	91.71 (199)	8.29 (18)
Conjunctivitis	0	100(211)*
Cough	71.43 (155)	28.57 (62)
Diarrhea	28.57 (62)	71.43 (155)
Fever	64.06 (139)	35.94 (78)
Low appetite	21.9 (46)	78.1 (164)*
Myalgia	35.19 (76)	64.81 (140)*
Nausea/vomiting	32.67 (136)	67.33 (136)*
Ageusia	8.61 (18)	91.39 (191)*
SOB	79.72 (173)	20.28 (44)*
Confusion	13.82 (30)	86.18 (187)*
Headache	22.12 (48)	77.88 (169)
Rhinorrhea	3.69 (8)	96.31 (209)
Sore throat	5.53 (12)	94.47 (205)
ACEI	36.41 (79)	63.59 (138)
CVD history	41.94 (91)	58.06 (126)
History of malignancy	8.29 (18)	91.71 (199)
History of DM	43.32 (94)	56.68 (123)
Received flu vaccine	45 (45)	55 (55)*
History of GI issues	24.42 (53)	75.58 (164)
History of HIV	0.92 (2)	99.08 (215)
History of HTN	70.97 (154)	29.03 (63)
NSAID usage	31.8 (69)	68.2 (148)
History of RT disease	39.17 (85)	60.83 (132)
History of smoking	38.25 (83)	61.75 (134)

 

**Figure 2:**
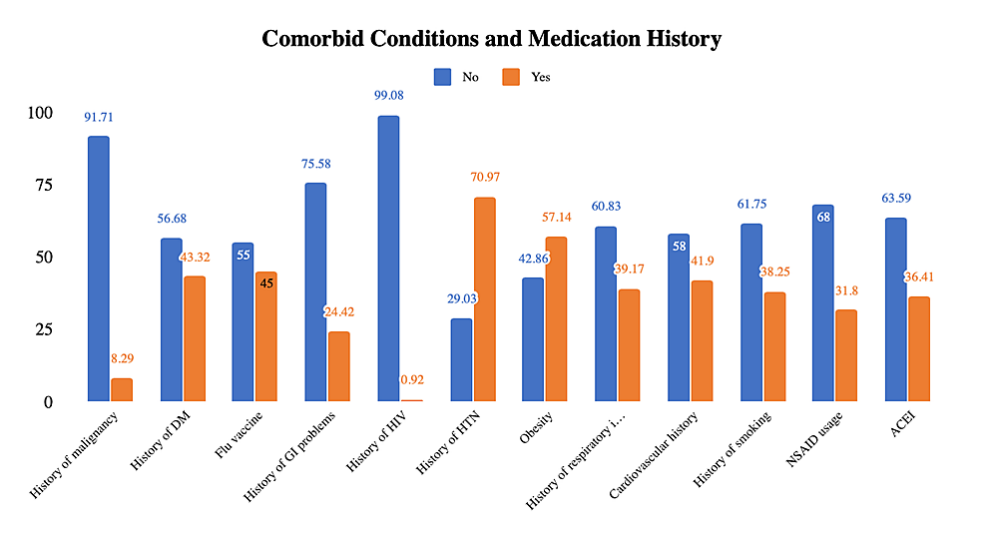
Comorbid conditions and medication history of patients admitted with COVID-19. DM: diabetes mellitus; GI: gastrointestinal; NSAID: nonsteroidal anti-inflammatory drug; HTN: hypertension; ACEI: angiotensin-converting enzyme inhibitor.

Comorbid conditions include diabetes mellitus (43%) of patients, history of flu vaccine (45%), gastrointestinal history (24.42%), HIV (0.92%), hypertension (HTN) (70.97%), nonsteroidal anti-inflammatory drug (NSAID) usage (31.8%), obesity (57.14%), angiotensin-converting enzyme inhibitor (ACEI) usage (36.4%), history of cardiovascular disease (41.94%), history of respiratory disease (39.17%), smoking history (38.25%), and cancer history (8.29%) (Table 1, Figure 2).

Outcome

Of the 217 people studied, 85% were discharged, 10.6% died, 3.69% were still in the hospital, and 0.46% were transferred to higher centers. When comparing clinical characteristics and mortality, age greater 63 was statistically significant (p-value: 0.0052), gender and chest pain were marginally significant with p-values of 0.0642 and 0.0944, respectively. The other variables investigated did not show any statistical significance. They were the following: anosmia (p-value: 0.2942), cough (p-value: 0.8343), fever (p-value: 0.7363), low appetite (p-value: 0.9215), myalgias (p-value: 0.6138), nausea/vomiting (p-value: 0.6840), ageusia (p-value: 0.138), SOB (p-value: 0.7159), confusion (p-value: 0.1637), headache (p-value: 0.6278), rhinorrhea (p-value: 0.3210), and sore throat (p-value: 0.2198) (Table 1).

When evaluating comorbid conditions and their association with mortality, HTN was statistically significant with a p-value of 0.0058, and there was marginal significance with cancer history (p-value: 0.0944). The other variables did not show statistical significance; and included: flu vaccine (p-value: 0.2688), NSAID use (p-value: 0.8820), CVD history (p-value: 0.1338), diabetes history (p-value: 0.3647), ACE-I use (p-value: 0.5291), GI history (p-value: 0.2214), HIV (p-value: 0.6247), obesity (p-value: 0.6105), history of RT disease (p-value: 0.6484), and smoking history (p-value: 0.1461) (Table 2).

**Table 2:** Clinical characteristics, comorbid conditions and their association with survival. *Statistically significant; **marginally significant. DM: diabetes mellitus; GI: gastrointestinal; NSAID: nonsteroidal anti-inflammatory drug; HTN: hypertension; CVD: cardiovascular disease; RT: respiratory tract; ACEI: angiotensin-converting enzyme inhibitor; SOB: shortness of breath.

Variables		Survivors (%) (n = 194)	Non-survivors (%) (n = 23)	p-value
Age	<63 years	95.33 (102)	4.67 (5)	0.0052*
	>63 years	83.64 (92)	16.36 (18)	
Gender	Females	93.04 (107)	6.96 (8)	0.0642*
	Males	85.29 (87)	14.71 (15)	
Anosmia	Yes	100 (9)	0 (0)	0.2942
	No	89.05 (179)	10.95 (22)	
Cough	Yes	89.68 (139)	10.32 (16)	0.8343
	No	88.71 (55)	11.29 (7)	
Chest pain	Yes	77.78 (14)	22.22 (4)	0.0944**
	No	90.45 (180)	9.55 (19)	
Fever	Yes	89.93 (125)	10.07 (14)	0.7363
	No	88.46 (69)	11.54 (9)	
Low appetite	Yes	89.13 (41)	10.87 (5)	0.9215
	No	89.63 (147)	10.37 (17)	
Myalgia	Yes	90.79 (69)	9.21 (7)	0.6138
	No	88.57 (124)	11.43 (16)	
Nausea/vomiting	Yes	89.39 (59)	10.61 (7)	0.6840
	No	91.18 (124)	8.82 (12)	
Ageusia	Yes	100 (18)	0	0.138
	No	89.01 (170)	10.99 (21)	
SOB	Yes	89.02 (154)	10.98 (19)	0.7159
	No	90.91 (40)	9.09 (4)	
Confusion	Yes	96.67 (29)	3.33 (1)	0.1637
	No	88.24 (165)	11.76 (22)	
Headache	Yes	87.50 (42)	12.50 (96)	0.6278
	No	89.94 (152)	10.06 (17)	
Rhinorrhea	Yes	100 (8)	0	0.3210
	No	89 (186)	11 (23)	
Sore throat	Yes	100 (12)	0	0.2198
	No	88.78 (182)	11.22 (23)	
Flu vaccine	Yes	91.11 (41)	8.89 (4)	0.2688
	No	3.64 (46)	16.36 (9)	
HTN	Yes	85.71 (132)	14.29 (22)	0.0058*
	No	98.41 (62)	1.59 (1)	
NSAID usage	Yes	89.86 (62)	10.14 (7)	0.8820
	No	89.19 (132)	10.81 (16)	
CVD history	Yes	85.71 (78)	14.29 (13)	0.1338
	No	92.06 (116)	7.94 (10)	
Cancer history	Yes	77.78 (14)	22.22 (4)	0.0944 **
	No	90.45 (180)	9.55 (19)	
Diabetes history	Yes	87.23 (82)	12.77 (12)	0.3647
	No	91.06 (112)	8.94 (11)	
ACEI	Yes	91.14 (72)	8.86 (7)	0.5291
	No	88.41 (122)	11.59 (16)	
GI history	Yes	84.91 (45)	15.09 (8)	0.2214
	No	90.85 (149)	9.15 (15)	
HIV	Yes	100 (2)	0	0.6247
	No	89.30 (192)	10.70 (23)	
Obesity	Yes	90.32 (112)	9.68 (12)	0.6105
	No	88.17 (82)	11.83 (11)	
History of RT disease	Yes	90.59 (77)	9.41 (8)	0.6484
	No	88.64 (117)	11.36 (15)	
Smoking history	Yes	85.54 (71)	14.46 (12)	0.1461
	No	91.79 (123)	8.21 (11)	

## Discussion

This is one of the first retrospective studies to summarize clinical features of COVID-19 patients in North West Ohio, comparing survivors and non-survivors. Our study found that patients with a history of HTN, cardiovascular disorders, and obesity were more commonly infected. When looking at symptomatology, chest pain was the most prominent symptom of COVID-19 infection with a prevalence of 91.71% but was also marginally associated with death (p-value: 0.0944). Besides, we found that most patients who did not survive were greater than 63 years old (p-value: 0.0052). Also, patients with HTN were at an increased risk of both infection and death (p-value: 0.0058). Similar studies have also shown this correlation between HTN and susceptibility to COVID-19 [10-12]. Surprisingly, males and those with a cancer history were associated with increased odds of death with marginally significant p-values of 0.0642 and 0.0944, respectively. 

When compared to other studies, we found a higher rate of chest pain in our patient population. Previous studies from China and Korea report a low rate of chest pain symptomatology in their patient population [13-15]. This is surprising and may be due to a late presentation of COVID-19 and subsequent late complications such as pericarditis and myocarditis [16]. There was an effort to discourage patients from coming to the hospital unless necessary to reduce their infection risk. It could be possible that many of those patients did not seek treatment until it was late into the course. Our data also showed CVD, HTN, and obesity associated with COVID-19 infection, similar to other U.S. studies [17,18].

In contrast to recent studies, however, our patient sample has a low prevalence of anosmia at 4.29%. In a recent study from Italy, 64% of symptomatic COVID-19 patients had anosmia, and again in a similar study from Iran, 98.3% of their infected patients had anosmia [19,20]. The mechanism for anosmia related to COVID-19 is not yet known. Still, a theory suggests cell disruption of the nasal epithelium, causing a proinflammatory state of the olfactory neuroepithelium that causes cell disruption to be the cause of anosmia [21]. Currently, we do not have any robust theories on why our patient population differs in this aspect of symptomatology. 

Another interesting symptom that was not as apparent in our study compared to others was the fever history. With 64.06% of our patient sample showing the clinical characteristic of fever, this is relatively low compared to other studies. Previous studies note over 85% of their patient sample with fever [17,22,23]. However, fever's presence did not appear to have any statistical significance compared to mortality risk (p-value: 0.7363). Lastly, G.I. symptoms such as nausea/vomiting (32.67%) and diarrhea (28.57%) were similarly low compared to previous studies in New York, the epicenter of COVID-19 in the United States and China [24]. We also did not notice a lot of central or peripheral neurological symptoms [25-27]. This suggests that coronavirus is primarily a respiratory-driven virus without much involvement of the GI tract. 

Accurately identifying the epidemiology and clinical characteristics of COVID-19 infection can help clinicians understand the disease course, susceptibility, and prognosis of patients with COVID-19 infection. Our findings in this study mostly correspond to prior studies indicating increased susceptibility for older patients, HTN, DM, and obesity. One interesting thing which we noted that, increased prevalence of chest pain with COVID-19 infection. And statistically, it is associated with increased mortality (with marginal significance).

Limitations

It is a retrospective study. The small sample size may not accurately depict the general population. Also, we cannot determine causation but only association. Lastly, it is difficult to rule out any confounders given the methodology of this study, and temporal relationships are challenging to assess. Larger studies are needed to identify mortality predictors, especially concerning the history of chest pain and cancer history.

## Conclusions

COVID-19 is a massive pandemic of the century, with more than 20 million infected and over 700,000 deaths. This retrospective cohort study investigates the epidemiology and characteristics of 217 patients of COVID-19 in Northwest Ohio, United States. Our findings show that age >63, male sex, HTN history, cancer history, and chest pain on admission may be significant predictors of COVID-19 outcomes.
